# Supplementary material for: Content-rich biological network constructed by mining PubMed abstracts
Source: BMC Bioinformatics. 2004 Oct 8;5:147. doi: 10.1186/1471-2105-5-147 (PMC528731; doi:10.1186/1471-2105-5-147)
Supplement: Additional File 2 — The original results of the above study (non-essential files are deleted to keep the file size under the limit set by BMC bioinformatics). [file 1471-2105-5-147-S2.bz2 › chilibotAdditionalFile2/dip40/372ID7730326E8295/html/FGF1_FGFR1.html]

 


 **FGF1** and **FGFR1** 
  
Found 826 abstracts in PubMed, retrieved 40.  
 

 What does Google say? 
 PDF only 
| .edu only 

---

**Interactive relationship** (e.g. stimulation, inhibition, etc)

**Inhibitory relationship**- Exogenous FGF  [ **FGF1** ]  2 decreased  **FGFR1**  protein.  Ref: 12746216 Am J Physiol Endocrinol Metab< MedlineTA>Am J Physiol Endocrinol Metab,
- Overexpression of mSef in NIH3T3 cells results in a decrease in FGF  [ **FGF1** ]  induced cell proliferation associated with a decrease in Tyr phosphorylation of  **FGFR1**  and FRS2.  Ref: 12604616 J Biol Chem, 2003
- These results indicate that Sef exerts its inhibitory effects at the level of FGFR  [ **FGFR1** ]  and upstream of Ras providing an additional level of negative regulation of FGF  [ **FGF1** ]  signaling.  Ref: 12604616 J Biol Chem, 2003
- Both the FGFR 1  [ **FGFR1** ]  inhibitor SU5402 and the MAPK pathway inhibitor PD98059 also significantly inhibited the activation of the MAPK pathway by FGF  [ **FGF1** ]  20 and the neurotrophic activity of FGF  [ **FGF1** ]  20.  Ref: 12704805 J Neurosci Res, 2003
**Neutral relationship**- Activated fibroblast growth factor receptor 1  [ **FGFR1** ]   **FGFR1**  propagates FGF  [ **FGF1** ]  signals through multiple intracellular pathways via intermediates FRS2, PLCgamma, and Ras.  Ref: 12621028 J Biol Chem, 2003
- FGF  [ **FGF1** ]  20 was also found to bind FGFR  [ **FGFR1** ]  1c with high affinity with the BIAcore system.  Ref: 12704805 J Neurosci Res, 2003
- Our studies have shown that full length FGF  [ **FGF1** ]  receptor 1  **FGFR1**  accumulates within the nuclear interior in parallel with FGF  [ **FGF1** ]  2.  Ref: 12647309 J Cell Biochem, 2003
- HSGAGs affect FGF  [ **FGF1** ]  signaling by interacting with both the growth factor and the FGF  [ **FGF1** ]  receptor FGFR  [ **FGFR1** ] .  Ref: 12659634 Biochem J< MedlineTA>Biochem J, 2003
- These findings suggest that  **FGFR1**  and FGFR2 in myofibroblasts may be responsible for the signal transduction of FGF  [ **FGF1** ]  during the wound healing process.  Ref: 12648559 Arch Oral Biol, 2003
- Keratinocyte growth factor receptor KGFR, also known as fibroblast growth factor receptor FGFR  [ **FGFR1** ]  2 IIIb, is located in many types of epithelial cells and is activated by four known ligands FGF 1  [ **FGF1** ] , FGF  [ **FGF1** ]  3, FGF  [ **FGF1** ]  7 also known as KGF and FGF  [ **FGF1** ]  10 that are predominantly synthesized by mesenchymal cells.  Ref: 12608893 Pathol Int, 2003
- Fibroblast growth factor FGF  [ **FGF1** ]  signaling begins with the formation of a ternary complex of FGF  [ **FGF1** ] , FGF  [ **FGF1** ]  receptor FGFR  [ **FGFR1** ] , and heparan sulfate HS.  Ref: 12604602 J Biol Chem, 2003
- However, Namalwa cells, immobilizing FGF  [ **FGF1** ]  2 via their cell surface CD44vRA, bound substantially more soluble FGF  [ **FGF1** ]  receptor 1 FGFR 1  [ **FGFR1** ]  than did Namalwa cells immobilizing the same amount of FGF  [ **FGF1** ]  2 via their cell surface CD44v3 v10.  Ref: 12697740 J Clin Invest, 2003
- Anchorage independent growth exhibited by Notch repressed NIH 3T3 cells may result from prolonged FGFR  [ **FGFR1** ]  stimulation caused by both an increase in the expression of prototypic and oncogenic FGF  [ **FGF1** ]  gene family members and the nonclassical export of  **FGF1**  into the extracellular compartment.  Ref: 12598523 J Biol Chem, 2003
- Hence, FGF  [ **FGF1** ]  2 attached to the heparan sulfate moiety expressed by the novel CD44 variant of RA synovium cells exhibits an augmented ability to stimulate FGFR 1  [ **FGFR1** ]  mediated activities.  Ref: 12697740 J Clin Invest, 2003

**Non-interactive relationship** (e.g. studied together, co-existance, homology, etc.)

- The objective of this study was to elucidate the correlation between the expression of FGF  [ **FGF1** ]  2, VEGF, and their receptors FGF  [ **FGF1** ]  receptor 1 FGFR 1  [ **FGFR1** ]  and VEGF receptor 1 Flt 1 with microvessel density in synovial tissues of the TMJ.  Ref: 12651930 J Dent Res, 2003
- The present findings indicate that the activation of the MAPK pathway by FGF  [ **FGF1** ]  20 signaling through FGFR  [ **FGFR1** ]  1c plays important roles in the survival of dopaminergic neurons in the SNPC.  Ref: 12704805 J Neurosci Res, 2003
- therefore, a model of 2 2 2  **FGF1** .HS. **FGFR1**  was shown to fit the data.  Ref: 12604602 J Biol Chem, 2003
- Colocalisation of  **FGFR1**  with FGF  [ **FGF1** ]  2 could not be demonstrated in unstressed retina, but was demonstrable in stressed retina, in both the ONL and OPL.  Ref: 12708794 Growth Factors, 2002
- Furthermore, the molar ratio of HS,  **FGF1** , and  **FGFR1**  in the ternary complex was found to be independent of the size of HS, which indicates that the selected model can take place on the cell surface proteoglycans.  Ref: 12604602 J Biol Chem, 2003
- Fibroblast growth factor FGF  [ **FGF1** ]  FGF  [ **FGF1** ]  receptor FGFR  [ **FGFR1** ]  signaling plays a crucial role in mesoderm formation and patterning.  Ref: 12893744 Circ ResCirc Res, 2003
- We also measured components of the FGF  [ **FGF1** ]  2 ternary signaling complex, fibroblast growth factor receptor 1  [ **FGFR1** ]  FGFR 1  [ **FGFR1** ]  and syndecan 4.  Ref: 12832283 Am J Physiol Lung Cell Mol Physiol< MedlineTA>Am J Physiol Lung Cell Mol Physiol, 2003
- The Involvement of Heparan Sulfate HS in  **FGF1**  HS  **FGFR1**  Signaling Complex.  Ref: 12604602 J Biol Chem, 2003
- We find that SMC and BAEC express the same FGFR  [ **FGFR1** ]  isoforms and bind FGF2 with equal affinity at the cell surface, yet FGF  [ **FGF1** ]  has a markedly higher proliferative effect on SMC than on BAEC.  Ref: 12659634 Biochem J< MedlineTA>Biochem J, 2003
- FGF  [ **FGF1** ]  2 alone stimulated maximal phosphorylation of Src homology domain 2 tyrosine phosphatase SHP 2 and the adaptor molecule Crk, in heparan sulfate deficient Chinese hamster ovary CHO 677 cells expressing FGFR 1  [ **FGFR1** ] .  Ref: 12799194 Exp Cell Res, 2003
- FGF10 and FGF  [ **FGF1** ]  receptor FGFR  [ **FGFR1** ]  2 immunostaining showed a similar epithelial expression pattern.  Ref: 12697038 J Endocrinol, 2003
- Thyroidal levels of fibroblast growth factor 2 FGF  [ **FGF1** ]  2 and fibroblast growth factor receptor 1  [ **FGFR1** ]   **FGFR1**  are elevated in human thyroid hyperplasia.  Ref: 12746216 Am J Physiol Endocrinol Metab< MedlineTA>Am J Physiol Endocrinol Metab, 2003
- To resolve the discrepancies, we measured the size and molar ratio of HS in the complex and showed that both  **FGF1**  and  **FGFR1**  simultaneously interact with HS.  Ref: 12604602 J Biol Chem, 2003
- The expression of FGF  [ **FGF1** ]  receptor FGFR  [ **FGFR1** ]  and TGF beta receptor was significantly higher in ALP positive PDLFs than in ALP negative PDLFs.  Ref: 12886987 J Periodontol, 2003
- Finally, a mechanism for the FGF  [ **FGF1** ] .FGFR  [ **FGFR1** ]  signaling complex formation on cell membrane was proposed, where FGF  [ **FGF1** ]  and FGFR  [ **FGFR1** ]  have their own binding sites on HS and a distinct ternary complex formation site is directly responsible for mitogenic activity.  Ref: 12604602 J Biol Chem, 2003
- FGF  [ **FGF1** ]  receptor FGFR  [ **FGFR1** ]  1c was found to be expressed abundantly in dopaminergic neurons in the SNPC but at much lower levels in neurons of other midbrain regions by in situ hybridization.  Ref: 12704805 J Neurosci Res, 2003
- We show that FGF  [ **FGF1** ]  signaling regulates Pyst1 expression in developing neural plate and limb bud by ablating and or transplanting tissue sources of FGFs and by applying FGF  [ **FGF1** ]  protein or a specific FGFR  [ **FGFR1** ]  inhibitor SU5402.  Ref: 12814546 Curr Biol, 2003
- Analysis of tryptic phosphopeptide maps of FGFR 1  [ **FGFR1** ]  from cells stimulated with FGF  [ **FGF1** ]  2 alone and FGF  [ **FGF1** ]  2 together with heparin showed that FGF  [ **FGF1** ]  2 alone stimulated a several fold increase in tyrosine 463 in the juxtamembrane domain.  Ref: 12799194 Exp Cell Res, 2003
- Interestingly, FGF  [ **FGF1** ]  exerts a negative effect on Notch by suppressing CSL CBF 1 RBP Jk KBF2 in mammals, Su H in Drosophila and Xenopus, and Lag 2 in Caenorhabditis elegans dependent transcription, and the ectopic expression of constitutively active forms of Notch1 or Notch2 abrogates  **FGF1**  release and the phenotypic effects of FGFR  [ **FGFR1** ]  stimulation.  Ref: 12598523 J Biol Chem, 2003
- Increases in  **FGFR1**  or FGF  [ **FGF1** ]  2 and both may contribute to goitrogenesis.  Ref: 12746216 Am J Physiol Endocrinol Metab< MedlineTA>Am J Physiol Endocrinol Metab, 2003
- Using genetic and biochemical methods, we generated HSs that were defective in  **FGF1**  and or  **FGFR1**  binding but could form the signaling ternary complex.  Ref: 12604602 J Biol Chem, 2003
- We have studied the mechanism whereby heparin directs FGF  [ **FGF1** ]  2 induced FGF  [ **FGF1** ]  receptor 1 FGFR 1  [ **FGFR1** ]  signal transduction.  Ref: 12799194 Exp Cell Res, 2003
- One of the best studied model systems for protein HSGAG interactions is the fibroblast growth factor FGF  [ **FGF1** ]  family of molecules, and recent observations have demonstrated that the specificity of a given FGF  [ **FGF1** ]  ligand binding to its cognate receptor FGFR  [ **FGFR1** ]  is mediated by distinct tissue specific HSGAG sequences.  Ref: 12604799 Proc Natl Acad Sci U S A, 2003
- Finally, isolated primary synovial fluid cells from RA patients expressing CD44vRA bound more soluble FGFR 1  [ **FGFR1** ]  to their cell surface associated FGF  [ **FGF1** ]  2 than did corresponding synovial cells expressing CD44v3 v10 or synovial cells from osteoarthritis patients.  Ref: 12697740 J Clin Invest, 2003
- These changes are associated with specific alterations in the regulation of FGF  [ **FGF1** ]  2, FGFR 1  [ **FGFR1** ]  and syndecan 4 in the airway epithelial mesenchymal trophic unit, which may be associated with the developmental problems of the lung associated with exposure to ozone.  Ref: 12832283 Am J Physiol Lung Cell Mol Physiol< MedlineTA>Am J Physiol Lung Cell Mol Physiol, 2003
- **FGFR1**  expression and  **FGFR1**  FGF  [ **FGF1** ]  2 colocalisation in rat retina sites of FGF  [ **FGF1** ]  2 action on rat photoreceptors.  Ref: 12708794 Growth Factors, 2002
- Here, we report that collateral artery growth in its early phase is associated with an increased expression of FGF  [ **FGF1** ]  receptor 1 FGFR 1  [ **FGFR1** ]  and syndecan 4 on mRNA and protein levels as well as with an increased kinase activity of FGFR 1  [ **FGFR1** ]  in a rabbit model of arteriogenesis.  Ref: 12600883 Circ Res, 2003
- Sensitivity to FGF  [ **FGF1** ]  2 disappeared after 7 days although  **FGFR1**  expression was maintained.  Ref: 12746216 Am J Physiol Endocrinol Metab< MedlineTA>Am J Physiol Endocrinol Metab, 2003
- METHODS Immunohistochemistry and confocal microscopy were used to localise FGF  [ **FGF1** ]  2 and  **FGFR1**  in cryosections of the rat retina, both normal retina and retina stressed by exposure to bright continuous light 1000 lux, 24h.  Ref: 12708794 Growth Factors, 2002
- We conclude that TSH stimulates  **FGFR1**  but not FGF  [ **FGF1** ]  2 expression.  Ref: 12746216 Am J Physiol Endocrinol Metab< MedlineTA>Am J Physiol Endocrinol Metab, 2002
- Northern and Western blot analysis and immunohistochemistry were used to screen for the fibroblast growth factor receptor 1  [ **FGFR1** ]  FGF  [ **FGF1** ]  R1 and its high affinity ligand, basic fibroblast growth factor bFGF, which could have a role in this recovery process.  Ref: 12626345 Am J Respir Crit Care Med, 2003
- This provides an initial description of the expression in the developing skull of the genes encoding the secreted molecules BMP 2, BMP 4, BMP 7, the transmembrane FGF  [ **FGF1** ]  receptors FGFR 1  [ **FGFR1** ] , FGFR  [ **FGFR1** ]  2, FGFR  [ **FGFR1** ]  4, the transcription factors Msx1, Msx2, and Twist, as well as Goosecoid and the early membranous bone differentiation marker osteopontin.  Ref: 12710966 Dev Biol, 2003
- Ligand distribution was correlated with FGF  [ **FGF1** ]  receptor FGFR  [ **FGFR1** ]  expression.  Ref: 12798286 Dev Biol, 2003
- Furthermore, mSef inhibits ERK activation mediated by a constitutively activated  **FGFR1**  but not by a constitutively active Ras and decreases FGF  [ **FGF1** ]  but not PDGF mediated activation of Akt.  Ref: 12604616 J Biol Chem, 2003
- FGF  [ **FGF1** ]  induced maturation of FGFR  [ **FGFR1** ]  expressing oocytes was blocked by previous injection of Grb7 or Grb14, but not Grb10.  Ref: 12885405 FEBS Lett, 2003
- Thus, FGF  [ **FGF1** ] , and 8, found in RTF, may act upon FGFR 1  [ **FGFR1** ]  IIIc in the principal cells of the initial segment to regulate GGT mRNA IV expression.  Ref: 12606343 Biol Reprod, 2003
- Colocalisation of  **FGFR1**  and FGF  [ **FGF1** ]  2 could be demonstrated in stressed retina, in the cytoplasm and the axon terminals of photoreceptors.  Ref: 12708794 Growth Factors, 2002
- Since the presence of FGF  [ **FGF1** ]  receptors FGFRs determines cellular responsiveness, we examined the localisation of  **FGFR1** , FGFR2 and FGFR3 expression by immunohistochemistry throughout the repair of full thickness excisional wounds up to 28 days after wounding.  Ref: 12648559 Arch Oral Biol, 2003
- To understand the significance of this, effects of  **FGFR1**  activation on normal human thyrocyte growth and function in vitro and the regulation of FGF  [ **FGF1** ]  2 and  **FGFR1**  expression have been examined.  Ref: 12746216 Am J Physiol Endocrinol Metab< MedlineTA>Am J Physiol Endocrinol Metab, 2003
- Tissue specific inactivation of one of the FGF  [ **FGF1** ]  receptor FGFR  [ **FGFR1** ]  genes,  **Fgfr1** , in the midbrain and rhombomere 1 of the hindbrain of mouse embryos results in deletion of the inferior colliculi in the posterior midbrain and vermis of the cerebellum.  Ref: 12682014 EMBO J, 2003
- The expression and potential roles of fibroblast growth factors FGF  [ **FGF1** ]  and their cognate FGF  [ **FGF1** ]  receptors FGFR  [ **FGFR1** ]  in adult mammalian retinal ganglion cells RGC are poorly known.  Ref: 12799136 Mol Cell Neurosci, 2003
- AIM To identify sites of FGF  [ **FGF1** ]  2 action on photoreceptors of the rat retina, by localizing  **FGFR1**  in the intact retina, and to assess the colocalisation of FGF  [ **FGF1** ]  2 with  **FGFR1** .  Ref: 12708794 Growth Factors, 2002
